# Cesarean scar pregnancy combined with arteriovenous malformation successfully treated with transvaginal fertility-sparing surgery: A case report and literature review

**DOI:** 10.1097/MD.0000000000021432

**Published:** 2020-07-31

**Authors:** Xiangjuan Li, Wenchao Sun, Lingna Chen, Mei Jin, Zhifen Zhang, Jiansong Gao, Xiaoyang Fei

**Affiliations:** a Department of Female Pelvic Medicine and Reconstructive Surgery; b Center of Reproductive Medicine; c Department of Gynecology; d Department of Ultrasonography, Hangzhou Women's Hospital, Hangzhou, China.

**Keywords:** arteriovenous malformation, cesarean scar pregnancy, fertility preservation, transvaginal surgery

## Abstract

**Introduction:**

A cesarean scar pregnancy (CSP), when combined with an arteriovenous malformation (AVM), is a rare, but potentially life-threatening condition that may be associated with uncontrolled hemorrhage. Hysterectomy is indicated when conservative treatment fails. Preservation of fertility is challenging.

**Patient concerns:**

We reported a 33-year-old woman with a CSP combined with an AVM who failed methotrexate administration as conservative treatment.

**Diagnoses:**

A CSP combined with an AVM was diagnosed via three-dimensional color Doppler angiogram and magnetic resonance imaging.

**Interventions:**

Transvaginal removal of the ectopic gestation and repair of the uterine defect was performed without incident.

**Outcomes:**

The fertility of the patient was preserved and hysterectomy was avoided.

**Conclusion:**

Transvaginal fertility-sparing surgery may be successfully performed to prevent hysterectomy when conservative treatment fails in patients with a CSP combined with an AVM.

**Teaching points**

- Although rare, an AVM can occur during CSP treatment and cause catastrophic hemorrhage, which makes conservative treatment difficult and can necessitate a hysterectomy.
- In patients with a CSP combined with an AVM, a hysterectomy can be avoided through the use of transvaginal fertility-sparing surgery with ectopic gestation removal and repair of the uterine defect, even when conservative treatment fails.
- Hemostasis techniques and separation skills, as adopted in female pelvic reconstructive surgery, can be used in the treatment of obstetric complications.

## 1 Introduction

Cesarean scar pregnancy (CSP) is a relatively new type of ectopic gestation in which the embryo is located in the scar from a previous cesarean delivery.^[1]^ The incidence of CSP is estimated to be 1 in 2226 pregnancies.^[2]^ In women who have had a cesarean delivery, 6.1% of ectopic gestations are CSPs.^[2]^ Since CSPs can cause serious maternal morbidity, such as uncontrolled hemorrhage and uterine rupture, early diagnosis and timely intervention are especially important.^[3]^ Thus far, greater than 30 treatment strategies for CSPs have been published, including resection via an abdominal, transvaginal, laparoscopic, or hysteroscopic approach, systemic or local methotrexate (MTX) injection, uterine artery embolization (UAE), and dilation and curettage (D&C).^[4]^

Of note, treatment of CSPs may lead to the development of an arteriovenous malformation (AVM).^[5]^ Uterine AVMs are characterized by abundant connections between arteries and veins in the uterus, which may lead to vaginal bleeding that may be severe.^[6]^ Uterine AVMs may be congenital or acquired. Acquired AVMs are usually the consequence of uterine instrumentation, such as a D&C or cesarean delivery.^[7]^ A causative connection between CSP treatment and acquired AVMs has recently been proposed.^[6]^ Whereas both CSPs and AVMs are potentially devastating, the combination of these 2 conditions may lead to catastrophic complications. Some patients require a hysterectomy after failed conservative treatment, resulting in loss of fertility.^[6,8]^

We present a patient with a CSP combined with an AVM who had acute heavy vaginal bleeding during conservative treatment and was successfully treated with transvaginal fertility-sparing surgery without the need for a hysterectomy.

## 2 Case report

A 33-year-old gravida 3 para 1 presented to the Reproductive Medicine Clinic complaining of light vaginal bleeding accompanied by paroxysmal lower abdominal pain, which had persisted for 21 days. Her medical history included a cesarean delivery 8 years ago due to oligohydramnios, and a CSP cured by UAE followed by a D&C 1 year ago. Twenty-four days before the presentation, she was diagnosed with a repeat CSP in a local hospital and underwent UAE followed by a D&C. The serum beta-human chorionic gonadotropin (β-hCG) level before UAE was 171,428 mIU/mL. The D&C, which was performed 3 days after the UAE, was complicated by massive bleeding that was controlled by continuous balloon compression for 24 hours. Products of conception were visible in the curettings. Ten days after the D&C, an ultrasound examination showed an abnormal echo at the uterine incision, approximately 4.4 × 3.9 × 3.0 cm^3^ in size. Mifepristone tablets were administered (50 mg orally twice a day for 3 days), but the β-hCG level did not decrease significantly and the vaginal bleeding did not stop. Surgical treatment was recommended, and the possibility of a hysterectomy was discussed. The patient had a strong desire for future fertility and was transferred to the Reproductive Center of our hospital. The patient has provided informed consent for publication of the case.

**Figure 1:**
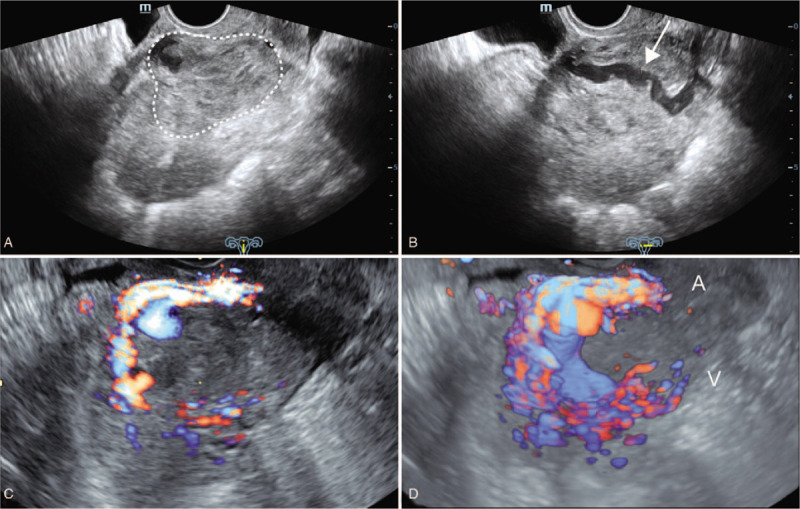
Transvaginal ultrasound image. A. Grayscale sagittal image showing a 5.4 × 5.0 cm^2^ lesion (*dashed circle*) at the anterior isthmic region of the uterus. B. Grayscale coronal image showing an echo-free tubular structure up to 1.0 cm in width within the lesion (*arrow*). C. Color Doppler imaging showing enhanced vascularity surrounding and within the lesion. D. Three-dimensional color Doppler angiogram showing the arteriovenous malformation and the feeding (*A*) and draining (*V*) vessels.

**Figure 2:**
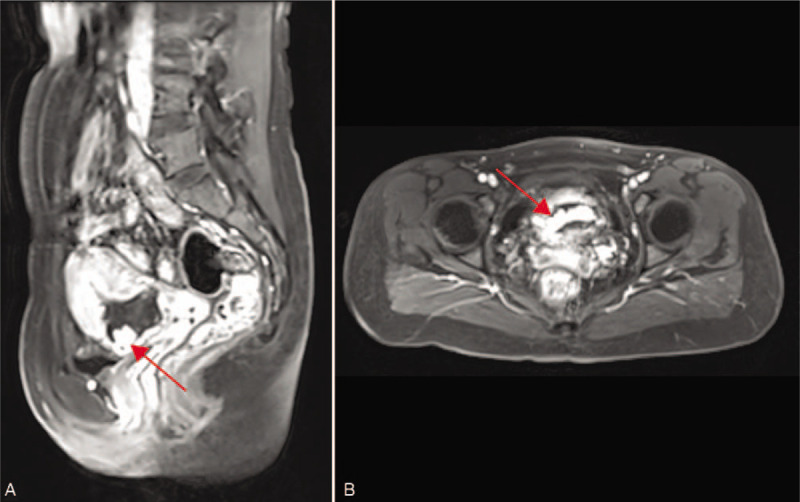
T1-weighted contrast-enhanced magnetic resonance imaging showing an enlarged vessel (*red arrow*) in the gestational mass, which is consistent with arteriovenous malformation. A. Sagittal view. B. Horizontal view.

At the current presentation, she was anemic with a hemoglobin level of 10.4 g/dL (reference range, 11.0–15.0 g/dL). The pulse was 67 beats per minute and the blood pressure was 120/76 mm Hg. The pelvic examination revealed a slightly enlarged, anteflexed, nontender uterus. A transvaginal gray scale ultrasound examination showed a lesion 5.4 × 5.0 cm^2^ in size at the lower anterior wall of the uterus (Fig. 1A, B). Color Doppler ultrasound showed dilated and tortuous blood vessels encompassing the lesion (Fig. 1C). An AVM in the CSP was suspected based on a three-dimensional color Doppler angiogram (Fig. 1D). The distance between the external cervix and the lower edge of the gestational sac was 4 cm. Magnetic resonance imaging findings were consistent with the AVM diagnosis (Fig. 2).

After a discussion with the patient, she expressed her appreciation for conservative management and agreed to systemic chemotherapy using MTX to preserve her fertility. A single dose of MTX (80 mg) was administered intramuscularly. The patient had intermittent light vaginal bleeding until an acute blood loss of 200 mL occurred on the 5th day after MTX administration. The hemorrhage was temporarily controlled by intravenous oxytocin and aminomethylbenzoic acid. A multidisciplinary discussion was held. Hysterectomy was discussed again, but physicians from the Department of Female Pelvic Medicine proposed transvaginal fertility-sparing surgery to simultaneously remove the ectopic gestation and repair the uterine defect, which would avert a hysterectomy and preserve fertility. The surgery was performed after signing an informed consent form.

**Figure 3:**
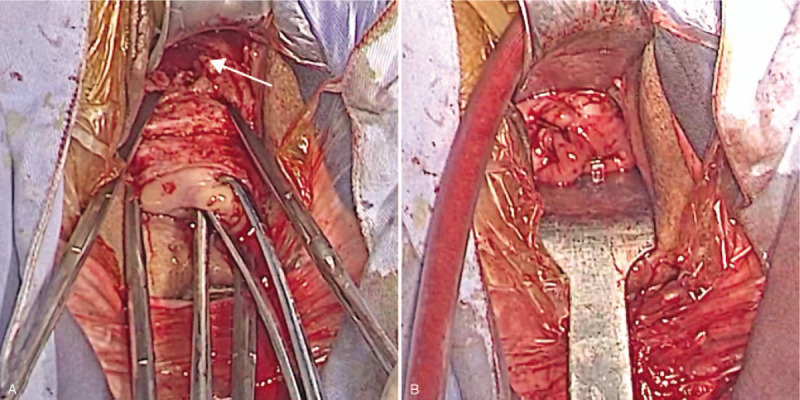
Transvaginal surgery. A. Ectopic gestation removed from the uterine defect (*arrow*). B. Uterine defect repaired.

**Figure 4:**
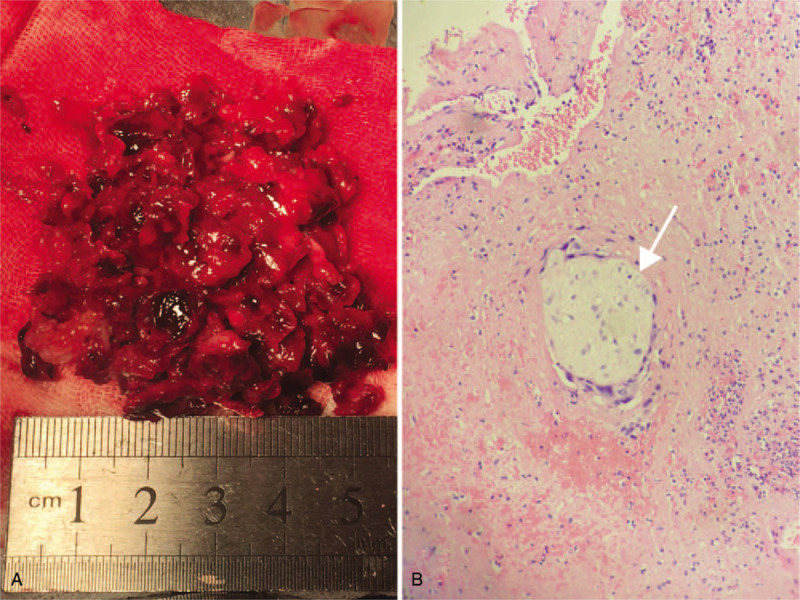
Surgical specimen. A. Ectopic gestation and blood clots removed from the anterioruterine segment. B. Photomicrograph of trophoblasts (*arrow*, hematoxylin and eosin stain, 40 × magnification).

The surgical procedure consisted of the following four steps. *Step 1. Exposure of the cervix.* After general anesthesia, the patient was placed in the dorsal lithotomy position and the bladder was emptied. A pair of vaginal retractors was placed into the vagina to expose the cervix. The cervix was then held on traction with 2 Jacobs tenacula. *Step 2. Dissecting the uterovesical space.* Diluted adrenaline (0.3 unit in 1 mL of normal saline [20 mL]) was injected submucosally to hydro-dissect the uterovesical space and achieve hemostasis. An incision was made at the junction of the vaginal mucosa and cervix. Using fingers, the bladder was bluntly separated away from the cervix, then sharply from the uterine isthmus using surgical scissors until the anterior peritoneal reflection was recognized. The CSP was identified as a thin, purple bulge at the anterior isthmic region of the uterus. *Step 3. Removal of the ectopic gestation.* Two curved Kelly clamps were placed immediately adjacent to the uterine cervix to block the descending branches of the bilateral uterine arteries and assure hemostasis. An arc incision was made over the most protuberant area of the bulge. The ectopic gestation and blood clots were removed with sponge forceps, and thorough curettage of the isthmus uteri was subsequently performed through the incision (Fig. 3A). The edges of the incision were carefully trimmed with scissors to ensure all of the scar tissue had been removed. *Step 4. Repair of the uterine defect.* The myometrial incisions were closed with 4 interrupted 1–0 sutures absorbable. The vaginal incisions were closed with continuous locking 2–0 absorbable sutures (Fig. 3 B). Iodoform gauze was placed in the vagina for the next 24 hours to avoid hematoma formation. The total estimated blood loss was 100 mL. The histologic examination confirmed the presence of trophoblasts (Fig. 4).

**Figure 5:**
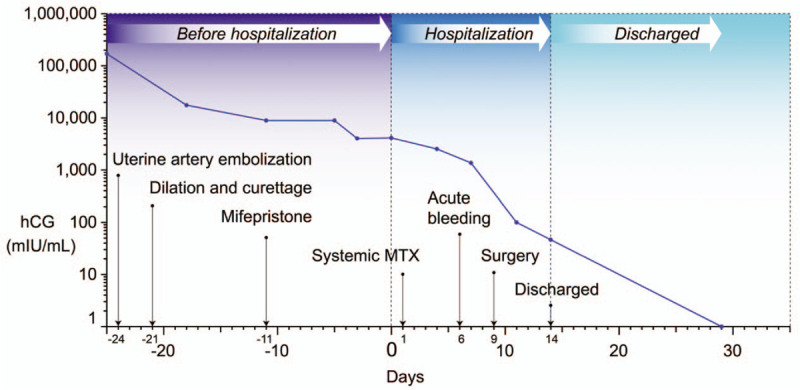
Kinetics of the serum β-hCG level and the corresponding treatment (day of hospitalization = day 0).

The postoperative recovery was uneventful. The β-hCG level dropped to 100.2 mIU/mL on postoperative day 2, and 46.6 mIU/mL on day 5. The patient was asymptomatic on postoperative day 5 and was discharged home. The β-hCG level dropped to < 5 mIU/mL 20 days after surgery (Fig. 5). Menstruation resumed 1 month after surgery. Eight months later the patient remained asymptomatic and the β-hCG level continued to be <5 mIU/mL.

## 3 Discussion

A 33-year-old woman with a CSP combined with an AVM who failed MTX administration as conservative treatment is described herein. Transvaginal removal of the ectopic gestation and repair of the uterine defect were successfully performed, and fertility was preserved. Fertility preservation is consistent with the concept of treatment for CSP.

Since the first report in 1978, CSP has become increasingly common worldwide.^[9]^ This finding may be attributed to the increase in cesarean delivery rates. The cesarean delivery rate in China has increased dramatically since the 1990s, with an alarming rate of 46.2% in 2007 to 2008.^[10]^ The high rate of cesarean delivery may be related to the One Child Policy in China; however, the policy was relaxed in November 2013 and many Chinese couples were allowed and subsequently encouraged to have a second child.^[11]^ Indeed, Chinese couples risked the occurrence of long-term complications of cesarean delivery,^[12]^ such as placenta previa, placenta accreta, uterine rupture, CSP, and even recurrent CSP (as in the present case), to attempt giving birth to a second child. The present case is a typical example of many Chinese women of childbearing age. Fertility preservation is extremely important and emphasized in all aspects of reproductive care in China and worldwide when treating CSP.^[4,13]^

Fertility preservation in patients with a CSP combined with an AVM is challenging. A co-existing CSP and AVM is a rare and potentially life-threatening combination that may be associated with uncontrolled hemorrhage, leading to hysterectomy. To ascertain the most effective treatment approach and subsequent fertility preservation outcomes to this rare combination, we searched the Medline/PubMed databases using the key words “cesarean scar pregnancy,” “uterine scar pregnancy,” and “arteriovenous malformation.” The search was limited to human subjects and English language for the period from January 1978 to March 2019. The starting point for the literature search was 1978 to coincide with the first reported case of CSP. Additional articles were retrieved from reference lists of relevant case reports and reviews. Our literature search yielded a total of 23 cases from 16 articles involving the management and outcomes of patients with a CSP complicated by an AVM (Table 1). The treatment modalities included conservative and surgical management. Conservative treatment was defined as treatment without abdominal or vaginal surgery. Surgical management included surgical resection of the lower uterine segment or a hysterectomy. Conservative treatment was initially applied to 21 of 23 patients (91.3%), 14 (60.9%) of who resolved without the need for additional surgery; however, when conservative treatment failed and additional surgery was needed, the hysterectomy rate was 71.4% (5/7), which was >2-fold the overall hysterectomy rate during treatment of CSPs complicated by AVMs (30.4%[7/23]). The incidence of hysterectomy was also higher than that of those who chose systemic MTX as a major conservative treatment for CSPs without AVMs (12.6%[29/230]).^[14]^ The patient presented herein failed MTX administration as conservative treatment, and therefore was at high risk for hysterectomy. She underwent a transvaginal hysterotomy as a fertility-sparing surgical procedure. Of note, the transvaginal removal of an ectopic gestation and repair of the uterine defect in treating a CSP combined with an AVM has not been previously reported.

**Table 1:**
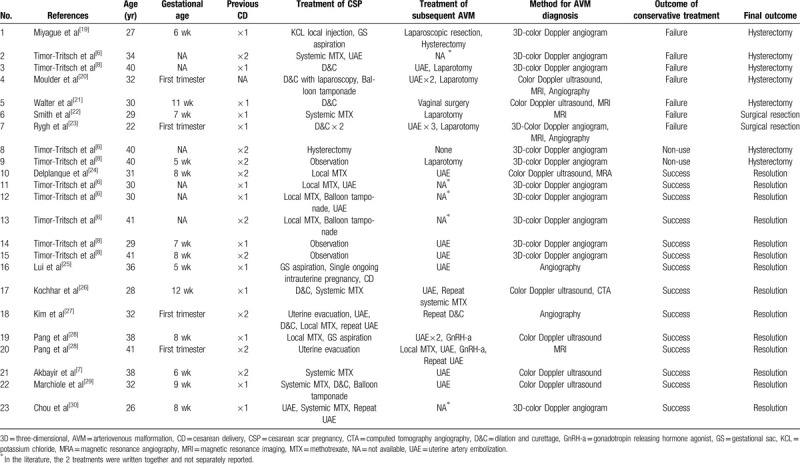
Summary of 23 CSPs combined with AVMs.

Transvaginal surgical management of CSPs without AVMs was first advocated by Kang et al in 2011,^[15]^ and has the following 5 advantages:

1. Transvaginal surgical management is the most minimally invasive method because the procedure is performed through a natural orifice.
2. The procedure is a shortcut to treat a CSP because the incision, made at the junction of the vaginal mucosa and cervix, has the least distance to the uterine isthmus, which allows the surgeon to find the lesion in the shortest time. This is an advantage for patients at risk of potentially major bleeding. Although UAE is effective in achieving hemostasis in the treatment of major bleeding, transvaginal surgical management can be a rescue therapy for CSP patients who have failed UAE treatment.^[16]^
3. Transvaginal surgical management requires only conventional equipment. For hospitals that do not offer UAE, the vaginal approach can be utilized as an alternative.
4. Hemostasis relies on suture rather than contraction of the uterine scar, which significantly reduces the risk of postoperative bleeding.^[17]^
5. The procedure allows the surgeon to repair the uterine defect at the same time as the pregnancy tissue is removed,^[16]^ which is beneficial for women who have the desired fertility because uterine integrity is retained.

Although transvaginal surgical management has the above advantages, surgical difficulty increases when the patient has a recurrent CSP combined with an AVM. The technical challenges include the following:

1. The vaginal approach has a narrow operating space and field of view, which can make achieving hemostasis comparatively difficult.
2. The previous 2 cesarean deliveries may cause strong adhesions between the bladder and ectopic gestation, making it difficult to separate the bladder from the cervix at the scar site. The risk of bladder injury also increases.
3. Cervical descent is impaired by dense adhesions, leading to an incomplete removal of the ectopic gestation.

There are three key points to overcome the above difficulties:

1. A series of measures were taken to reduce bleeding, including the submucosal injection of diluted adrenaline before dissecting the uterovesical space, and placement of 2 curved Kelly clamps adjacent to the uterine cervix before removal of the ectopic gestation.
2. Transvaginal surgery begins with an incision at the cervicovaginal junction, which makes separation of the bladder from the cervix start from the normal uterovesical space (relatively easy) and end at the scar site adhesions (more difficult). At the uterovesical adhesion site, sharp separation close to the uterus under direct vision can avoid damage to the bladder. The difficulty with surgery in this patient was no greater than transabdominal surgery because the surgery was performed by an experienced urogynecologist who was familiar with the anatomy of the pelvic floor and bladder.
3. The distance between the external cervix and the lower edge of the gestational sac should be measured by vaginal ultrasound before surgery to determine the degree of difficulty with cervical descent during surgery. The distance in the patient was 4 cm, which is within the normal value of the distance between the uterine isthmus to the external cervix (3.5–4 cm).^[18]^ Therefore, under general anesthesia, it is not difficult to achieve cervical descent with traction to the vaginal opening. After mastering these 3 key points, techniques involved in female pelvic reconstructive surgery may be used to treat obstetric complications and achieve good therapeutic results.

## Acknowledgments

The authors are grateful to the doctors and staff who have been involved in this work.

## Author contributions

**Conceptualization:** Wenchao Sun.

**Data curation:** Xiangjuan Li, Lingna Chen, Mei Jin.

**Investigation:** Xiangjuan Li, Lingna Chen, Jiansong Gao.

**Project administration:** Xiaoyang Fei.

**Software:** Mei Jin.

**Supervision:** Zhifen Zhang.

**Visualization:** Xiaoyang Fei.

**Writing – original draft:** Xiangjuan Li, Wenchao Sun.

**Writing – review & editing:** Zhifen Zhang, Xiaoyang Fei.
